# Complete genome of *Pseudomonas* sp. PAMC25886, a polar-adapted biodegrader from alpine glacier cryoconite

**DOI:** 10.1128/mra.00245-26

**Published:** 2026-05-18

**Authors:** Ji-Young Lee, Byeollee Kim, So-Ra Han, Yung Mi Lee, Sung Gu Lee, Jun Hyuck Lee, Tae-Jin Oh

**Affiliations:** 1 Department of Pharmaceutical Engineering and Biotechnology, SunMoon University, Asan, Republic of Korea; 2 Genome-Based BioIT Convergence Institute, Asan, Republic of Korea; 3 Division of Life Sciences, Korea Polar Research Institute, Incheon, Republic of Korea; DOE Joint Genome Institute, Berkeley, California, USA

**Keywords:** *Pseudomonas* sp. PAMC25886, high-quality assembly, polar-adapted, biodegrading organism

## Abstract

We determined the complete genome sequence of *Pseudomonas* sp. PAMC25886 from alpine glacier cryoconite. The genome comprises a single circular chromosome of 7,035,536 bp with 61.2% G + C content and 6,409 protein-coding genes. This genome provides insight into aromatic compound degradation and cold adaptation in polar environments.

## ANNOUNCEMENT

*Pseudomonas* sp. PAMC25886, originally isolated from alpine glacier cryoconite, was previously reported only with a fragmented draft genome consisting of multiple scaffolds and contigs, limiting comprehensive genomic analysis, which showed potential for pollutant degradation and biofilm formation (1). Polar *Pseudomonas* strains are relatively rare, and their genetic mechanisms for survival in extreme environments remain poorly understood. Here, we present a complete genome assembly, overcoming previous limitations and providing insights into genes for aromatic compound degradation, cold adaptation, and stress tolerance, highlighting the strain’s potential as a polar-adapted biodegrader.

*Pseudomonas* sp. PAMC25886 was obtained from the Korea Polar Research Institute, originally isolated from alpine glacier cryoconite in the Alps permafrost region (1). A single colony was cultured on R2A agar at 15°C, and genomic DNA was extracted using a DNeasy Blood & Tissue Kit (Qiagen, Netherlands). The same DNA extract was used for both Illumina and Oxford Nanopore Technologies (ONT) library preparation. The 16S rRNA gene was amplified with universal primers 27F/1492R (Cosmo Genetech, Korea) and sequenced using Sanger sequencing. Taxonomic identity was confirmed via EzBioCloud and NCBI BLAST, with the highest similarity to *Pseudomonas kielensis* (accession no. NR_181570.1) (2, 3).

Whole-genome sequencing was performed using Illumina and ONT platforms. Illumina libraries were prepared with the TruSeq DNA Nano Kit and sequenced on a NovaSeq 6000 (2 × 150  bp paired end), generating 17,977,679 reads (~357× coverage). ONT libraries were prepared using the 1D Ligation Sequencing Kit (SQK-LSK110) and the Native Barcoding Expansion Kit (EXP-NBD104) and sequenced on a GridION with FLO-MIN106 (R9.4) flow cells using high-accuracy basecalling on the GridION platform. ONT sequencing produced 37,640 reads (624.9 Mb; N50 = 24,545  bp; ~133× coverage). Reads were filtered using Filtlong and trimmed using fastp prior to assembly.

Following Wick et al. (4), long reads were assembled and polished with long and short reads. Assembly was performed using Trycycler (v0.5.4) (5) for consensus generation, Flye (v2.9.2) (6) for initial assembly, Medaka (v1.12.1) (7) for long-read polishing, and Polypolish (v0.6.0) (8) and POLCA (v0.3.1) (9) for short-read polishing. Contigs were circularized in Trycycler (10). Assembly quality was evaluated using QUAST (v5.2.0) (11) and CheckM (v1.2.2) (12). Taxonomic identity was confirmed through OrthoANI-based average nucleotide identity (ANI) analysis using reference genomes obtained from the EzBioCloud and NCBI databases (13). Gene annotation was conducted with RAST (14), KEGG (15), and eggNOG-mapper (16). All tools were run with default parameters unless otherwise specified.

The assembled genome, summarized in Table 1, comprises a single circular chromosome of 7,035,536 bp with 61.2% G + C content, encoding 6,409 protein-coding sequences, 66 tRNAs, and 16 rRNAs. ANI analysis indicated the highest similarity (99.93%) to *Pseudomonas* sp. NC02 (accession no. CP025624), while comparison with validly published species showed the closest similarity to *Pseudomonas kielensis*. The genome harbors multiple cold-adaptation genes, including six cold shock proteins (CSP family), GroEL, and DnaK chaperones, as well as enzymes involved in aromatic compound degradation, such as protocatechuate 3,4-dioxygenase (alpha and beta chains), homoprotocatechuate degradative operon repressors, and catalase KatE, highlighting its potential as a polar-adapted biodegrader.

**TABLE 1:** Genome features of the *Pseudomonas* sp. PAMC25886 genome sequence

Genome feature	PAMC 25886
Illumina	
Number of reads	17,977,679
Number of bases	5,393,303,700
Coverage	~357×
Oxford Nanopore	
Number of reads	37,640
Number of bases	624,900,000
Coverage	~133×
Genome size (bp)	7,035,536
G + C content (%)	61.2
Total number of genes	6,665
Number of CDSs (total)	6,409
Number of rRNAs (5S, 16S, 23S)	16
Number of tRNAs	66

## Data Availability

This *Pseudomonas* sp. PAMC25886 genome project has been submitted to the European Nucleotide Archive (ENA) under study accession number PRJEB106067 and sample accession number SAMEA121008658. The complete genome sequence has been deposited in GenBank under accession number OZ405150.1, with the assembly accessible under GCA_978045425.1. The raw sequencing reads have been deposited to the ENA under ERR16145642 and ERR17019655.
